# Xanthone synthetic derivatives with high anticandidal activity and positive mycostatic selectivity index values

**DOI:** 10.1038/s41598-023-38963-4

**Published:** 2023-07-23

**Authors:** Kamila Rząd, Rachel Ioannidi, Panagiotis Marakos, Nicole Pouli, Mateusz Olszewski, Ioannis K. Kostakis, Iwona Gabriel

**Affiliations:** 1 Department of Pharmaceutical Technology and Biochemistry, Faculty of Chemistry and BioTechMed Center, Gdańsk University of Technology, 11/12 Narutowicza Str., 80-233 Gdańsk, Poland; 2 Division of Pharmaceutical Chemistry, Department of Pharmacy, School of Health Sciences, National and Kapodistrian University of Athens, Panepistimiopolis, 15771 Zografou, Greece

**Keywords:** Drug discovery, Microbiology, Chemistry

## Abstract

With the current massive increases in drug-resistant microbial infection as well as the significant role of fungal infections in the death toll of COVID-19, discovering new antifungals is extremely important. Natural and synthetic xanthones are promising derivatives, although only few reports have demonstrated their antifungal mechanism of action in detail. Newly synthetized by us xanthone derivative **44** exhibited strong antifungal activity against reference and fluconazole resistant *C. albicans* strains. Our results indicate that the most active compounds **42** and **44** are not substrates for fungal ABC transporters (Cdr1p and Cdr2p) and Mdr1p, the main representative of the major facilitator superfamily efflux pumps, membrane proteins that are responsible for the development of resistance. Moreover, fungicidal mode of action reduces the probability of persistent or recurrent infections and resistance development. In this light, the demonstrated killing activity of the examined derivatives is their undoubted advantage. Novel synthesized compounds exhibited moderate cytotoxicity against human cell lines, although the selectivity index value for human pathogenic strains remained favourable. Our results also indicate that novel synthetized compounds **42** and **44** with antifungal activity target yeast topoisomerase II activity. In summary, further validation of xanthones applicability as antifungals is highly valuable.

## Introduction

Fungal microorganisms are etiological factors of severe, often deadly, infectious diseases, especially in immunocompromised patients. The number of these patients is growing rapidly, not only because of diseases resulting in immunodeficiency, like AIDS but also as a consequence of the frequent use of therapies that affect the human immune defense system (e.g.anticancer therapy with cytostatics, steroid therapy, use of the immunosuppressive agents in transplant patients). Systemic mycoses are caused in these patients mainly by yeast-like microorganisms from the *Candida* genus, especially *Candida albicans* and *Candida glabrata*, and filamentous fungi from the *Aspergillus* genus^1^. On the other hand, many fungal microorganisms are known as one of the most frequent reasons of nosocomial infections. *C. albicans* is considered the fourth most popular etiological agent of nosocomial infections worldwide. Moreover, chemotherapeutics used in clinical treatment have become factors stimulating the selection of resistant cells. A newly described pathogen, *Candida auris*, is an emerging multidrug-resistant organism that poses a global threat^2^. Additionally, invasive fungal infections complicate the clinical course of COVID-19 and are associated with a significant increase in mortality, especially in critically ill patients admitted to an intensive care unit^3^. Thus, with the current massive increases in drug-resistant microbial infections as well as the significant role of fungal infections in the death toll of COVID-19, discovering new antifungal compounds is extremely important.

There are several approaches in novel drug discovery. First of all, researchers are looking for new drugs targeting old pathways (e.g., ergosterol synthesis)^4^ or cell membranes^5^, while others are trying to find out new solutions. The biosynthesis of fungal proteins, DNA, and other essential molecules is extremely important^6,7^. As long as new targets are concerned, our group is looking for new drugs targeting fungal topoisomerases. Significant work has been done on the structure and function of topoisomerase I and II in fungi and results indicated that their activities are crucial for some specific strains^8–10^. Moreover, inhibition of yeast topoisomerase II resulted in antifungal activity^11,12^ and even managed to overcome fluconazole-resistance^13,14^.

Natural xanthone derivatives are a promising group of antifungal compounds^15–17^. They are present in nature as metabolites of various plant, lichen, fungal, and bacterial species^18,19^. The interesting structural scaffold and biological efficacy of those compounds lead many scientists to synthesize xanthone derivatives for the development of new prospective drug candidates as anticancer, antimicrobial, antimalarial, anti-HIV, antioxidant, anti-inflammatory, and antimalarial agents^20^. Several articles have been published highlighting the antifungal activity of synthetic xanthone analogues^15,21–23^, although only a few have been examined thoroughly in order to define their mechanism of action. According to previous reports, 1,2-dihydroxyxanthone is the most active compound against all fungal strains tested, showing its effect on sterol biosynthesis by reducing the amount of ergosterol detected^24^.

Emerging from previous studies performed for xanthone analogues as potential antimicrobials^15–24^, we have decided to analyze the antifungal activity of four new groups of compounds. As xanthone derivatives with anticancer activity are reported to be effective human topoisomerase inhibitors^20^, we have also analyzed the inhibitory effect of selected derivatives on yeast topoisomerase II activity (yTOPO II).

## Results and discussion

### Synthesis of xanthone derivatives

**Figure 1:**
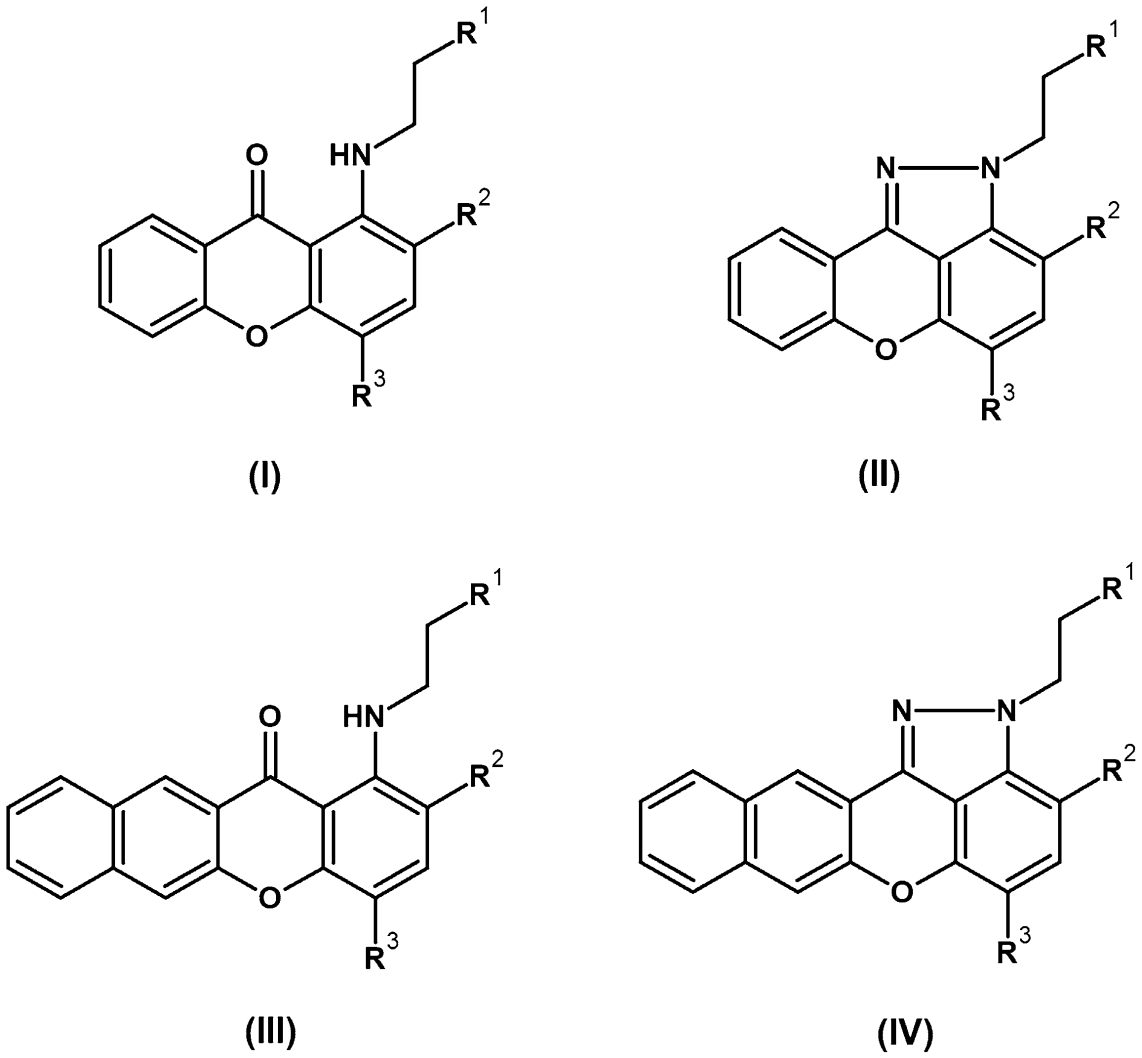
Overall structures of derivatives that were analysed in this study. R^1^—aminosubstitution, R^2^ or R^3^—NO_2_ or H.

The structures of xanthone derivatives synthesized in this study are divided into four groups (Fig. 1). Compounds **1**–**16** and **34** were prepared according to a previously published procedure^25,26^, while **25**, **26**, **35–38**, and **41–45** are newly synthesized analogues (Table 1). These groups (I–IV) differ in the nitro or amino substitution and the presence of a pyrazole or a benzene fused ring on the xanthone core (Fig. 1).

**Table 1:** The list of xanthone (group I and II) and benzoxanthone (group III and IV) analoques.

Compound	R^1^	R^2^	R^3^
Group (I)			
** 1**	N(CH_2_CH_3_)_2_	H	NO_2_
** 2**	N(CH_2_CH_3_)_2_	H	H
** 3**		H	NO_2_
** 4**		H	H
** 25**	N(CH_2_CH_3_)_2_	NO_2_	H
** 26**		NO_2_	H
Group (II)			
** 5**		H	H
** 6**		H	NO_2_
** 7**	N(CH_2_CH_3_)_2_	H	NO_2_
** 8**	N(CH_3_)_2_	H	H
Group (III)			
** 9**		H	NO_2_
** 10**	N(CH_2_CH_3_)_2_	H	NO_2_
** 11**	N(CH_3_)_2_	H	H
** 34**		NO_2_	H
** 35**	OCH_2_CH_2_OH	H	NO_2_
** 36**	CH_2_OH	H	NO_2_
** 37**	OH	H	NO_2_
** 38**	CH_2_OCH_3_	H	NO_2_
** 41**		H	NO_2_
** 42**		H	NO_2_
** 43**		H	NO_2_
** 44**		H	NO_2_
** 45**		H	NO_2_
Group (IV)			
** 12**		H	H
** 13**	N(CH_2_CH_3_)_2_	H	NO_2_
** 14**		H	NO_2_
** 15**		H	H
** 16**	N(CH_2_CH_3_)_2_	H	NO_2_

The list of xanthone and benzoxanthone analogues analyzed in this study is presented in Table 1.

**Figure 2:**
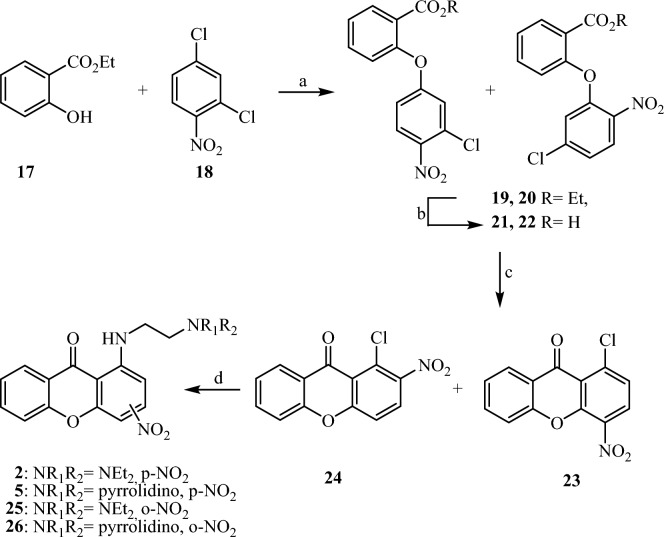
Reaction and conditions: (**a**) K_2_CO_3_, Cu_2_O, DMF dry, 110 °C; (**b**) NaOH 40%, EtOH, rt; (**c**) PPA, 110 °C; (**d**) suitable amine, pyridine, reflux.

**Figure 3:**
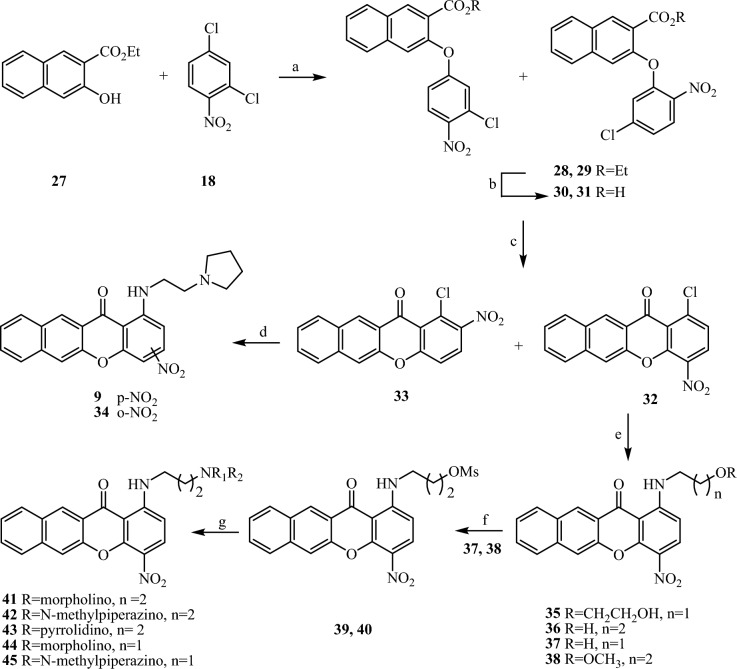
Reaction and conditions: (**a**) K_2_CO_3_, Cu_2_O, DMF dry, 110 °C; (**b**) NaOH 40%, EtOH, rt; (**c**) PPA, 110 °C; (**d**) suitable amine, pyridine, reflux; (**e**) suitable amine, pyridine, reflux; (**f**) MsCl, Et_3_N, THF, rt; (**g**) suitable amine, EtOH, reflux.

For the synthesis of the new compounds we have used an analogous synthetic procedure, with slight alterations (Figs. 2 and 3). Briefly, the reaction of ethyl salicylate (**17**) or ethyl 3-hydroxy-2-napthoate (**27**) with 2,4-dichloronitrobenzene (**18**) afforded a mixture of the isomeric diarylethers **19**, **20** and **28**, **29** respectively. Trituration of both mixtures with methanol resulted in pure **20** and **29**, while we obtained a 1/1 mixture of **19**, **20**, and **28**, **29**, which could not be further purified . For the synthesis of the ortho-substituted amines **25**, **26** and **34**, each of the above mixtures was saponified under mild conditions. Without further purification, the resulting mixture of acids **21**, **22**, and **30**, **31**, was ring closed upon treatment with polyphosphoric acid (PPA) to afford nitro compounds **23**, **24,** and **32**, **33** respectively, as inseparable mixtures. Reaction of the above mixtures with the suitable amines resulted in the aminoderivatives **2**, **5**, **25**, **26**, and **9**, **34** respectively. Each aminoderivative was isolated in pure form by column chromatography and identified by means of ^1^H and ^13^C spectral data, using both direct and long-range experiments (HMBC and HMQC). In order to prepare the corresponding p-substituted nitro derivatives **35–38** and **41–45**, ethyl ester **29** was saponified and ring closed upon treatment with PPA to afford the nitrosubstituted benzoxanthone **32** in pure form. Consequently, the amino derivatives **35–38** were prepared upon nucleophilic substitution of the chloro group of **32** by the appropriately substituted amines. For the synthesis of amines **41**–**45**, compounds **37** and **38** were converted to the mesylates **39** and **40**, which were treated with the appropriately substituted amines to result in the amino derivatives **41**–**43** and **44**–**45**, respectively.

### Susceptibility testing of novel compounds against fungal strains

All 28 derivatives were tested for their in vitro antifungal activity against five reference fungal strains (from American Type Culture Collection, ATCC) (Table 2). The most active 13 compounds were tested against six *C. albicans* clinical isolates, sensitive (B3, Gu4 and F2) and resistant (B4, Gu5 and F5) to fluconazole^27,28^ (Table 3). Minimal inhibitory concentrations (MICs) of the studied compounds were determined by the microplate serial dilution method^29^.

**Table 2:** Antifungal activity against reference strains. MIC_90_, minimal inhibitory concentration—a concentration that inhibits 90% of fungal cell growth.

Compound	*MIC_90_ µg mL^−1^				
	*Candida albicans* ATCC 10231	*Candida glabrata* ATCC 90030	*Candida krusei* ATCC 6258	*Candida parapsilosis* ATCC 22019	*Saccharomyces cerevisiae* ATCC 9763
**1**	32	32	32	> 64	16
**2**	> 64	> 64	64	32	32
**3**	16	16	16	64	8
**4**	> 64	> 64	64	64	64
**5**	16	> 64	32	32	32
**6**	16	8	16	> 64	8
**7**	16	16	16	> 64	16
**8**	32	64	32	32	16
**9**	4	4	4	8	4
**10**	> 16	> 16	> 16	> 16	> 16
**11**	> 64	> 64	> 64	> 64	> 64
**12**	8	> 64	8	> 64	8
**13**	8	4	4	16	2
**14**	8	4	4	16	4
**15**	8	32	8	64	4
**16**	32	16	8	32	16
**25**	> 64	> 64	> 64	64	64
**26**	> 64	> 64	64	64	64
**34**	32	> 32	8	> 32	8
**35**	> 64	> 64	> 64	> 64	> 64
**36**	> 64	> 64	> 64	> 64	> 64
**37**	> 64	> 64	> 64	> 64	> 64
**38**	> 64	> 64	> 64	> 64	> 64
**41**	> 64	> 64	> 64	> 64	> 64
**42**	8	4	4	8	4
**43**	> 64	> 64	> 64	> 64	> 64
**44**	4	2	2	4	2
**45**	32	32	32	32	32
**Amphotericin B**	0.5	1	1	1	0.5

* > Means no activity at the concentration mentioned. In this assay, the MIC_90_ value of amphotericin B was recorded as a positive control. The experiments were performed in three replicates.

**Table 3:** Antifungal activity of selected derivatives against clinical strains in comparison with *C. albicans* ATCC 10231.

Compound	*MIC_90_ µg mL^−1^						
	*Candida albicans* ATCC 10231	*Candida albicans* B3	*Candida albicans* B4	*Candida albicans* Gu4	*Candida albicans* Gu5	*Candida albicans* F2	*Candida albicans* F5
**3**	16	16	32	16	32	16	32
**5**	16	64	64	64	64	32	64
**6**	16	16	64	16	64	32	32
**7**	16	32	64	32	32	32	32
**8**	32	32	32	32	32	32	32
**9**	4	8	8	8	8	8	8
**12**	8	64	64	64	64	> 64	> 64
**13**	8	16	16	16	16	16	16
**14**	8	16	16	8	8	8	8
**15**	8	8	8	8	8	8	8
**42**	8	8	8	8	8	8	8
**44**	4	4	4	4	4	4	4
**45**	32	64	64	64	64	64	64
**Fluconazole**	8	1	16	4	> 64	8	> 64

* > Means no activity at the concentration mentioned. In this assay, the MIC_90_ value of fluconazole was recorded as a positive control. The experiments were performed at least in three replicates.

As reported in Table 2, the most active against reference strains from group III is derivative **9**, and from group IV compounds **13–15**, although it depends on the strain for the latter. Structure activity relationship analysis revealed that the presence of the naphthalene ring as well as the nitro group at R^3^ position (Fig. 1) is crucial for the antifungal activity. Thus, we decided to synthesize additional derivatives of compound **9** (**35–38** and **41–45**). This approach allowed us to obtain a derivative with even better antifungal activity (**44**) against reference strains than starting derivative **9**.

For the most active compounds, MIC_90_ was also determined against six *C. albicans* clinical isolates, sensitive (B3, Gu4, and F2) and resistant (B4, Gu5, and F5) to fluconazole.

The FLU-resistant clinical *C. albicans* B4, Gu5 and F5 strains were sensitive to all selected by us derivatives (Table 3). For some of these derivatives at the same level as their FLU-sensitive counterparts B3, Gu4 and F2 while for other MICs are higher for FLU-resistant cells but still measurable. The most active against reference strains compounds: **9** as well as **42**, **44** and **13**–**15** also exhibited the highest antifungal activity against clinical strains, including those FLU-resistant. Interestingly, antifungal activity of compound **15** against reference strains *C. glabrata* as well as *C. parapsilosis* is not significant (Table 2) but was found to be very active against clinical *C. albicans* strains, both sensitive and resistant. Strains Gu4, B3 and F2 are fluconazole-sensitive isolates obtained from early infection episodes, while Gu5, B4 and F5 are the corresponding fluconazole-resistant isolates obtained from later episodes in the same patients treated with fluconazole^27,28^. In the case of Gu5, the lack of susceptibility to fluconazole is a consequence of overexpression of CDR1/2 genes encoding ABC transporters, whereas the resistance of B4 and F5 strain is caused by overexpression of MDR1 gene encoding a membrane transport protein of the major facilitator superfamily (MFS)^27,28^. Our results indicate that the most active compounds **9**, **13**–**15**, **42** and **44** are not substrates for pumps that efflux drugs from resistant cells and their antifungal activity does not depend on the type of overexpressed pumps.

### Molecular mechanism of antifungal activity

#### Killing activity of selected derivatives

To establish the possible mode of action of xanthone and benzoxanthone analogues we have analyzed the killing activity and determined the minimal fungicidal concentrations (MFCs) for selected derivatives (Table 4).

**Table 4:** Fungicidal activity of selected compounds and amphotericin B (MFC—minimal fungicidal concentration—a concentration that inhibits 99% of fungal cell growth).

Compound	*MFC µg mL^−1^			
	*Candida albicans* ATCC 10231	*Candida glabrata* ATCC 90030	*Candida krusei* ATCC 6258	*Saccharomyces cerevisiae* ATCC 9763
**1**	64	> 128	> 128	16
**2**	> 64	> 64	> 64	64
**3**	32	128	64	16
**4**	> 64	> 64	> 64	64
**5**	64	> 128	128	32
**6**	32	128	64	8
**7**	64	> 128	128	16
**8**	64	> 64	64	32
**9**	8	8	8	4
**10**	> 16	> 16	> 16	> 16
**11**	> 64	> 64	> 64	> 64
**12**	> 64	> 64	> 64	> 64
**13**	32	32	8	16
**14**	16	16	8	8
**15**	64	64	32	16
**16**	64	32	32	32
**25**	> 64	> 64	> 64	> 64
**26**	> 64	> 64	> 64	> 64
**34**	> 32	> 32	32	32
**42**	8	8	4	4
**44**	4	4	4	2
**45**	32	32	32	32
**Amphotericin B**	2	2	2	2

* > Means no 99% killing activity at the concentration mentioned. Higher concentrations have not been tested due to the solubility limitations of some compounds. In this assay, the MFC value of amphotericin B was recorded as a positive control. The experiments were performed at least in three replicates.

Our results indicate that the mode of action of the most active compounds is fungicidal. The higher fungicidal activity was determined for derivatives **9, 42** and **44** from group III and **14** from group IV. The use of fungicidal therapy of invasive candidiasis and candidemia is associated with a higher probability of early therapeutic success. A decreased probability of persistent or recurrent infection and resistance development is also expected. In addition, frequent use of available fungistatic drugs like fluconazole promotes drug resistance^30^. In this light, the demonstrated killing activity of the examined derivatives is their undoubted advantage.

#### Inhibition of the relaxation activity of yeast topoisomerase II in vitro

**Figure 4:**
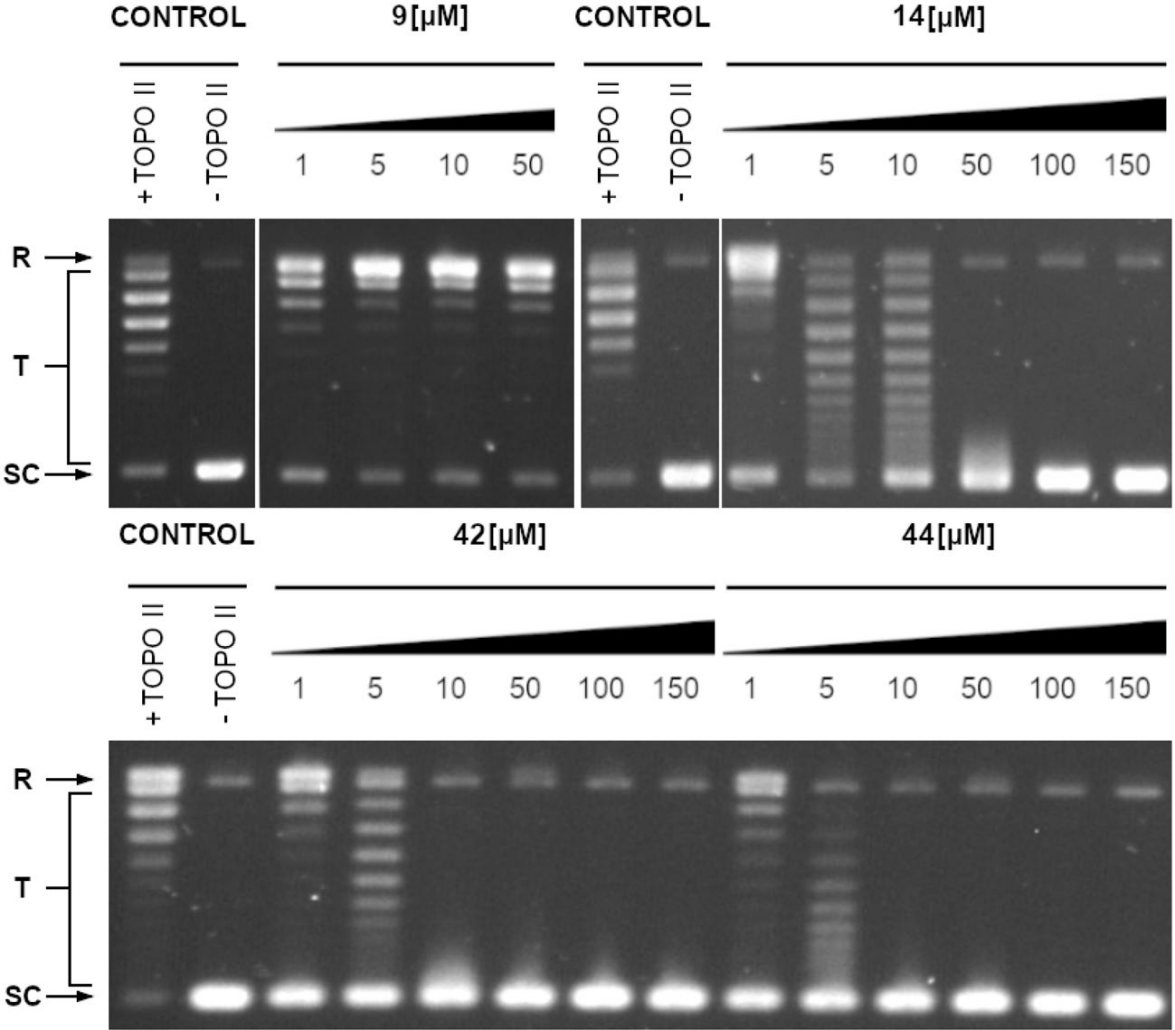
Inhibition of the catalytic activity of purified yeast DNA topoisomerase II by compounds **9**, **14**, **42** and **44** as measured by relaxation. Supercoiled pBR322 plasmid DNA (- TOPO II) was relaxed by purified yeast topoisomerase II in the absence (+ TOPO II) or presence of analyzed compounds **14**, **42** and **44** at 1–150 μM concentrations or 1–50 μM for **9** (higher concentrations have not been tested due to the low solubility of the compound). The resulting topological forms of DNA were separated by gel electrophoreses. SC, supercoiled DNA; R, relaxed DNA; T, DNA topoisomers. Data shown are typical of three independent experiments and one set of representative pictures are shown. Original gels are presented in Supplementary Fig. S1.

The xanthone derivatives act as anticancer agents through several mechanisms of action. The most important are the activation of caspase proteins and the inhibition of protein kinases and topoisomerases^31^. Due to known activity of xanthone derivatives as human topoisomerase II inhibitors^20,31^, we decided to evaluate the influence of the six more active compounds on the fungal equivalent of that enzyme. The effect of selected compounds on yeast topoisomerase II-mediated relaxation activity suggests their molecular target (Table 5 and Fig. 4).

**Table 5:** The concentrations of selected compounds that totally inhibited yeast topoisomerase II-mediated relaxation activity.

Compound	Complete inhibition [µM]	IC_50_ [µM]
9	> 50*	–
13	> 150	–
14	50	18.62 ± 1.81
15	> 150	–
42	10	5.86 ± 1.07
44	10	5.36 ± 1.22

For selected compounds IC_50_—inhibitory concentrations representing a concentration that inhibits 50% of enzyme activity were determined. The experiments were performed at least in three replicates.

*Higher concentrations have not been tested due to the low solubility of the compound.

As reported in Table 5, the most effective inhibitors towards fungal topoisomerase II are compounds **42** and **44**. Surprisingly, no inhibition was observed in the tested concentration range for compound **9**, the closely related to **42** and **44** derivatives. Thus, the molecular mode of action may vary between these compounds and needs more in depth analysis.

### Selectivity in relation to mammalian cells

Selected compounds were screened for their in vitro antiproliferative activity towards a human embryonic kidney cell line (HEK-293) and human liver cancer cell line (HEPG2) using colorimetric MTT assay. Our results were compared with the cytotoxic activity determined previously for human colorectal adenocarcinoma cell line (HT29)^26^ and are presented in Table 6.

**Table 6:** Determination of cytotoxic effect using MTT assay towards HEK293 and HEPG2 cell lines.

Compound	HEK293	HEPG2	HT29
**9**	3.64 ± 0.23	1.94 ± 0.67	–
**13**	5.73 ± 1.64	1.92 ± 0.85	1.85 ± 0.21*
**14**	5.99 ± 1.05	4.2 ± 0.71	1.83 ± 0.46*
**15**	8.33 ± 0.75	2.53 ± 0.82	10.7 ± 3.25*
**42**	5.38 ± 0.35	4.88 ± 0.31	–
**44**	3.57 ± 0.25	4.94 ± 0.56	–
**45**	48.47 ± 1.50	26.64 ± 2.36	–
**Etoposide**	1.91 ± 0.97	5.01 ± 0.22	–
**Doxorubicin**	6.52 ± 0.13**	–	–

In vitro growth inhibitory activity of compounds presented as an EC_50_ ± SD (µM) value—a concentration that inhibits 50% of mammalian cell growth.

*Antiproliferative activity of compounds **13–15** published previously^26^.

**Antiproliferative activity of doxorubicin published previously^32^. In this assay, the EC_50_ value of etoposide was recorded as a positive control.

As indicated for etoposide and doxorubicin^32^, both chemotherapy drugs used to treat different types of cancers, results obtained for newly synthezised compounds are within acceptable cytotoxicity levels. To estimate the selectivity in relation to mammalian cells mycostatic selectivity index (MSI) for *C. albicans* ATCC 10231 was calculated as the ratio of EC_50_ to MIC_90_ values after converting the MIC_90_ value to micromolar concentrations. The results are presented in Table 7.

**Table 7:** Mycostatic selectivity index values determined for *C. albicans* ATCC 10231 in relation to mammalian cell lines HEK293 and HEPG2.

Compound	EC_50_HEK293/MIC_90_	EC_50_HEPG2/MIC_90_
**9**	0.400	0.213
**13**	0.314	0.105
**14**	0.327	0.229
**15**	0.408	0.124
**42**	0.300	0.272
**44**	0.375	0.518
**45**	0.655	0.360

Positive mycostatic selectivity index values (MSI = EC_50_/MIC_90_) were obtained for all selected derivatives, ranging from 0.105 to 0.655. Our results indicate the possibility of using xanthone derivatives as efficient antifungal agents. However, it is necessary to improve their selectivity. Compound **44** is the most interesting, for which a higher level of MSI using HEPG2 cells was obtained. The HEPG2 cell line, derived from human liver cancer, is the most commonly used in studies on drug metabolism and toxicity^33^.

## Material and methods

### Chemical synthesis

#### General experimental procedures

All commercially available reagents and solvents were purchased from Alfa Aesar and used without any further purification. Melting points were determined on a Büchi apparatus and were uncorrected. All NMR spectra were recorded on 400 or 600 MHz Bruker spectrometers respectively Avance™ DRX and III instruments (Bruker BioSpin GmbH–Rheinstetten, Germany). ^1^H NMR (400 and 600 MHz) and ^13^C NMR (101 and 151 MHz, recorded with complete proton decoupling) spectra were obtained with samples dissolved in CDCl_3_ or DMSO‐*d6* with the residual solvent signals used as internal references: 7.26 ppm for CHCl_3_, and 2.50 ppm for (CD_3_)(CD_2_H)S(O) regarding ^1^H NMR experiments; 77.2 ppm for CDCl_3_ and 39.4 ppm for (CD_3_)_2_S(O) concerning ^13^C NMR experiments. Chemical shifts (*δ*) are given in ppm to the nearest 0.01 (1H) or 0.1 ppm (^13^C). The coupling constants (*J*) are given in Hertz (Hz). The signals are reported as follows: (s = singlet, d = doublet, t = triplet, m = multiplet, br = broad). Assignments of ^1^H and ^13^C NMR signals were unambiguously achieved with the help of D/H exchange and 2D techniques: COSY, NOESY, HMQC, and HMBC experiments. Compounds **1–8, 10–16** and **34** were synthesized according to literature and their ^1^H NMR and ^13^C NMR were compared with those reported into the literature^25,26^. Flash chromatography was performed on Merck silica gel (40–63 μm) with the indicated solvent system using gradients of increasing polarity in most cases (Merck KGaA—Darmstadt, Germany). The reactions were monitored by analytical thin-layer chromatography (Merck pre-coated silica gel 60 F254 TLC plates, 0.25-mm layer thickness). Compounds were visualized on TLC plates by both UV radiation (254 and 365 nm). All solvents for absorption and fluorescence experiments were of spectroscopic grade. Mass spectra were recorded on a UPLC Triple TOF–MS {UPLC:Acquity of Waters (USA), SCIEX Triple TOF–MS 5600 + (USA)}.

##### General procedure for the synthesis of amino substituted xanthones** 25** and** 26**

A solution of ethyl salicylate (1.66 g, 10 mmol, **17**), 2,4-dichloronitrobenzene (1.91 g, 9.95 mmol, **18**), K_2_CO_3_ (1.38 g, 10 mmol) and Cu_2_O (142 mg, 1 mmol) in dry DMF (10 mL) was heated at 110 °C for 12 h, under an argon atmosphere. After completion of the reaction the mixture was filtered hot, washed with EtOAc and the filtrate was vacuum evaporated. The oily residue was diluted in CH_2_Cl_2_, washed with water, dried over anh. Na_2_SO_4_ and evaporated to dryness. The obtained oily residue was triturated with hot ethanol (15 mL) with stirring and after cooling was filtrated to afford 1.0 g (36%) of **20** and 1.2 g (44%) of an oily mixture of esters **19** and **20** which was used for the next step without any further purification. To a suspension of the above mixture in ethanol (10 mL) a cold 40% NaOH solution was added and the resulting mixture was stirred at room temperature for 40 min. After completion of the reaction the mixture was poured into ice-water and acidified with 18% HCl solution. The resulting mixture of acids **21** and **22** was filtered, dried over P_2_O_5_ and dissolved in hot polyphosphoric acid. The resulting mixture was stirred at 110 °C for 1 h and upon cooling was poured into ice, and the precipitate was filtered and dried over P_2_O_5_ to afford xanthones **23** and **24** which were used for the next step without any further purification. Thus, **23** and **24** and the suitable amine, in dry pyridine, was refluxed for 1 1/2 h. After completion of the reaction, pyridine was vacuum evaporated, the oily residue was diluted in EtOAc, washed with water, dried over anh. Na_2_SO_4_ and evaporated to dryness. Flash chromatography on silica gel using a mixture of CH_2_Cl_2_/MeOH 93/7 as the elouent afforded the title compounds **25**, **26** and their isomers **2**^26^ and **5**^26^, respectively.

##### Data for 1-((2-(diethylamino)ethyl)amino)-2-nitro-9*H*-xanthen-9-one (**25**)

Yield: 12%; M.p. 123–124 °C (EtOAc–*n*-Hexane); ^1^H NMR (400 MHz, CDCl_3_) δ 10.89 (s br, D_2_O exchang., 1H), 8.26 (dd, *J* = 7.9, 1.7 Hz, 1H), 8.09 (d, *J* = 9.2 Hz, 1H), 7.69 (td, *J* = 7.9, 1.7 Hz, 1H), 7.46–7.36 (m, 2H), 6.57 (d, *J* = 9.3 Hz, 1H), 3.03 (q, *J* = 5.7 Hz, 2H), 2.72 (t, *J* = 5.7 Hz, 2H), 2.65 (q, *J* = 7.1 Hz, 4H), 1.08 (t, *J* = 7.1 Hz, 6H). ^13^C NMR (400 MHz, CDCl_3_) δ 179.0, 161.0, 155.1, 148.2, 135.2, 134.3, 130.6, 126.8, 125.2, 122.2, 117.8, 109.8, 104.0, 52.1, 47.2, 45.4, 12.0. ( +) ESI QqToF (m/z): Calcd. for C_19_H_22_N_3_O_4_^+^: [M + H]^+^ 356.1605, found 356.1614.

##### Data for 2-nitro-1-((2-(pyrrolidin-1-yl)ethyl)amino)-9*H*-xanthen-9-one (**26**)

Yield: 9%; M.p. 173–175 °C (EtOAc–n-Hexane); ^1^H NMR (400 MHz, CDCl_3_) δ 10.88 (t, D_2_O exchang., *J* = 4.7 Hz, 1H), 8.21 (dd, *J* = 7.9, 1.7 Hz, 1H), 8.06 (d, *J* = 9.3 Hz, 1H), 7.68 (td, *J* = 8.6, 1.7 Hz, 1H), 7.45–7.32 (m, 2H), 6.56 (d, *J* = 9.3 Hz, 1H), 3.13 (q, *J* = 6.3 Hz, 2H), 2.84 (t, *J* = 6.3 Hz, 2H), 2.66 (m, 4H), 1.85 (m, 4H). ^13^C NMR (400 MHz, CDCl_3_) δ 178.9, 160.5, 154.6, 147.6, 134.9, 133.9, 130.1, 126.3, 124.8, 121.6, 117.4, 109.2, 103.7, 54.9, 54.0, 46.0, 23.7. ( +) ESI QqToF (m/z): Calcd. for C_19_H_20_N_3_O_4_^+^: [M + H]^+^ 354.1448, found 354.1440.

##### 2-nitro-1-((2-(pyrrolidin-1-yl)ethyl)amino)-12*H*-benzo[*b*]xanthen-12-one (**34**)

This compound was prepared by an analogous procedure as described for **26** starting from ethyl 3-hydroxy-2-napthoate (**27**) with 2,4-dichloronitrobenzene (**18**). Flash chromatography on silica gel using a mixture of CH_2_Cl_2_/MeOH 94/6 as the elouent afforded the title compound **34**, and the para nitro substitutent compound **9**^26^. Yield: 12%; M.p. > 220 °C (EtOAc); ^1^H NMR (400 MHz, CDCl_3_) δ 10.99 t, D_2_O exchang., *J* = 6.0 Hz, 1H), 8.85 (s, 1H), 8.17 (d, *J* = 9.4 Hz, 1H), 8.08 (d, *J* = 8.3 Hz, 1H), 7.93 (d, *J* = 8.3 Hz, 1H), 7.86 (s, 1H), 7.66 (t, *J* = 8.3 Hz, 1H), 7.55 (t, *J* = 8.3 Hz, 1H), 6.73 (d, *J* = 9.4 Hz, 1H), 3.31 (q, *J* = 6.5 Hz, 2H), 3,03 (t, *J* = 6.5 Hz, 2H), 2.88 (m, 4H), 1.96 (m, 4H). ^13^C NMR (400 MHz, CDCl_3_) δ 180.0, 161.2, 150.6, 148.0, 136.8, 134.5, 130.3, 130.2, 129.8, 129.6, 128.3, 127.2, 126.2, 120.6, 113.4, 108.6, 105.5, 54.0, 43.3, 23.5. ( +) ESI QqToF (m/z): Calcd. for C_23_H_22_N_3_O_4_^+^: [M + H]^+^ 401.1605, found 401.1616.

##### General procedure for the synthesis of amino substituted benzo[*b*]xanthones** 35–38**

A solution of pure benzo[*b*]xanthone **32**, obtained from the synthesis of **34,** and the suitable amine (× 10 equiv.) in dry pyridine (5 mL) was refluxed for 2 h. After completion of the reaction, pyridine was vacuum evaporated, the oily residue was diluted in EtOAc, washed with water, dried over anh. Na_2_SO_4_ and evaporated to dryness. Flash chromatography on silica gel, using a mixture of CH_2_Cl_2_/MeOH 100/1 as the elouent provided the title compounds **35**–**38**.

##### Data for 1-((2-(2-hydroxyethoxy)ethyl)amino)-4-nitro-12*H*-benzo[*b*]xanthen-12-one (**35**)

Yield: 91%; Oil; ^1^H NMR (600 MHz, CDCl_3_) δ 10.95 (t, *J* = 5.0 Hz, 1H), 8.77 (s, 1H), 8.32 (d, *J* = 9.5 Hz, 1H), 8.01 (d, *J* = 8.3 Hz, 1H), 7.99 (s, 1H), 7.92 (d, *J* = 8.3 Hz, 1H), 7.62 (t, *J* = 8.2 Hz, 1H), 7.51 (t, *J* = 8.1 Hz, 1H), 6.42 (d, *J* = 9.5 Hz, 1H), 3.88 (m, 4H), 3.76–3.73 (m, 2H), 3.59 (q, *J* = 5.2 Hz, 2H). ^13^C NMR (600 MHz, CDCl_3_) δ 180.1, 155.9, 154.0, 150.7, 136.9, 134.1, 130.4, 129.8, 129.5, 127.8, 127.5, 126.3, 125.5, 120.9, 114.3, 104.8, 103.4, 72.9, 68.6, 62.1, 42.8. ( +) ESI QqToF (m/z): Calcd. for C_21_H_19_N_2_O_6_^+^: [M + H]^+^ 395.1238, found 395.1248.

##### Data for 1-((3-hydroxypropyl)amino)-4-nitro-12*H*-benzo[*b*]xanthen-12-one (**36**)

Yield: 84%; M.p. > 220 °C (EtOAc); ^1^H NMR (400 MHz, CDCl_3_) δ 10.78 (s, 1H), 8.77 (s, 1H), 8.33 (d, *J* = 9.6 Hz, 1H), 8.02 (d, *J* = 9.9 Hz, 2H), 7.93 (d, *J* = 8.4 Hz, 1H), 7.63 (t, *J* = 8.2 Hz, 1H), 7.52 (t, *J* = 8.2 Hz, 1H), 6.50 (d, *J* = 9.7 Hz, 1H), 3.91 (t, *J* = 5.8 Hz, 2H), 3.57 (q, *J* = 6.2 Hz, 2H), 2.07 (m, 2H). ^13^C NMR (400 MHz, CDCl_3_) δ 180.0, 156.1, 154.0, 150.7, 136.8, 134.0, 130.3, 129.8, 129.5, 127.6, 127.5, 126.3, 125.3, 120.9, 114.3, 104.6, 103.4, 60.3, 40.3, 31.5. ( +) ESI QqToF (m/z): Calcd. for C_20_H_16_N_2_O_5_Na^+^: [M + Na]^+^ 387.0951, found 387.0962.

##### Data for 1-((2-hydroxyethyl)amino)-4-nitro-12*H*-benzo[*b*]xanthen-12-one (**37**)

Yield: 89%; M.p. > 220 °C (EtOH-H_2_O); ^1^H NMR (400 MHz, CDCl_3_, MeOD) δ 8.75 (s, 1H), 8.30 (d, *J* = 8.4 Hz, 1H), 8.01 (d, *J* = 8.4 Hz, 1H), 7.97 (s, 1H), 7.91 (d, *J* = 8.4 Hz, 1H), 7.61 (t, *J* = 8.4 Hz, 1H), 7.50 (t, *J* = 8.3 Hz, 1H), 6.53 (d, *J* = 9.7 Hz, 1H), 3.89 (t, *J* = 5.6 Hz, 2H), 3.54 (t, *J* = 5.6 Hz, 2H). ^13^C NMR (400 MHz, CDCl_3_, MeOD) δ 180.4, 156.7, 154.5, 151.0, 137.3, 134.4, 130.8, 130.1, 129.9, 128.0, 127.9, 126.7, 125.5, 121.3, 114.6, 105.0, 104.2, 60.4, 45.7. (-) ESI QqToF (m/z): Calcd. for C_19_H_13_N_2_O_5_^−^: [M—H]^−^ 349.0830, found 349.0825.

##### Data for 1-((3-methoxypropyl)amino)-4-nitro-12*H*-benzo[*b*]xanthen-12-one (**38**)

Yield: 94%; M.p. 174–176 °C (EtOAc); ^1^H NMR (400 MHz, CDCl_3_) δ 10.76 (s, 1H), 8.78 (s, 1H), 8.32 (d, *J* = 9.7 Hz, 1H), 8.03 (d, *J* = 8.4 Hz, 1H), 8.00 (s, 1H), 7.93 (d, *J* = 8.4 Hz, 1H), 7.63 (t, *J* = 8.3 Hz, 1H), 7.52 (t, *J* = 8.1 Hz, 1H), 6.48 (d, *J* = 9.7 Hz, 1H), 3.57 (t, *J* = 5.7 Hz, 2H), 3.55–3.49 (m, 2H), 3.41 (s, 3H), 2.05 (m, 2H). ^13^C NMR (400 MHz, CDCl_3_) δ 179.9, 156.1, 154.0, 150.7, 136.8, 134.0, 130.3, 129.8, 129.4, 127.6, 127.5, 126.2, 125.3, 121.0, 114.2, 104.6, 103.4, 69.9, 59.0, 40.6, 29.2. ( +) ESI QqToF (m/z): Calcd. for C_21_H_18_N_2_O_5_Na^+^: [M + Na]^+^ 401.1108, found 401.1115.

##### General procedure for the synthesis of amino substituted benzo[*b*]xanthones **41–43** and** 44–45**

Methanesulfonyl chloride (82 μL, 1.05 mmol) was added dropwise at 0 °C to a suspension of **36** (1 mmol) or **37** (1 mmol) and Et_3_N (279 μL, 2 mmol) in dry CH_2_Cl_2_ (10 mL) and the resulting mixture was stirred at room temperature for 6 h. After completion of the reaction the mixture was washed with 1N HCl (2 × 5 mL) and water (2 × 5 mL) and the organic layer was dried over Na_2_SO_4_ and vacuum evaporated. Without further purification, the obtained crude mesylate (**39** and **40** respectivelly), was dissolved in absolute ethanol (10 mL) and to this solution a suitable amine (× 10 equiv.) was added. The resulting solution was stirred under reflux for 12 h. The solvent was then vacuum evaporated and the oily residue was purified by flash chromatography on silica gel, using a mixture of CH_2_Cl_2_/MeOH 9/1 as the elouent to provide the title compounds **41**–**43** and **44**–**45**.

##### Data for 1-((3-morpholinopropyl)amino)-4-nitro-12*H*-benzo[*b*]xanthen-12-one (**41**)

Yield: 77%; M.p. 179–180 °C (EtOAc–*n*-Hexane); ^1^H NMR (400 MHz, CDCl_3_) δ 10.73 (t, D_2_O exchang., *J* = 5.5 Hz, 1H), 8.73 (s, 1H), 8.30 (d, *J* = 9.6 Hz, 1H), 8.00 (d, *J* = 8.4 Hz, 1H), 7.97 (s, 1H), 7.91 (d, *J* = 8.4 Hz, 1H), 7.62 (t, *J* = 8.2 Hz, 1H), 7.50 (t, *J* = 8.2 Hz, 1H), 6.47 (d, *J* = 9.7 Hz, 1H), 3.77 (m, 4H), 3.47 (q, *J* = 6.4 Hz, 2H), 2.59–2.45 (m, 6H), 1.96 (p, *J* = 6.4 Hz, 2H). ^13^C NMR (400 MHz, CDCl_3_) δ 179.7, 155.9, 153.8, 150.5, 136.6, 133.8, 130.2, 129.6, 129.3, 127.4 (2 C), 126.1, 125.1, 120.8, 114.1, 104.4, 103.3, 67.0, 56.0, 53.8, 41.3, 25.7. ( +) ESI QqToF (m/z): Calcd. for C_24_H_24_N_3_O_5_^+^: [M + H]^+^ 434.1710, found 434.1717.

##### Data for 1-((3-(4-methylpiperazin-1-yl)propyl)amino)-4-nitro-12*H*-benzo[b]xanthen-12-one (**42**)

Yield: 81%; Oil; ^1^H NMR (400 MHz, CDCl_3_) δ 10.74 (t, D_2_O exchang., *J* = 5.4 Hz, 1H), 8.73 (s, 1H), 8.30 (d, *J* = 9.6 Hz, 1H), 8.06–8.00 (d, *J* = 9.6 Hz, 1H), 7.96 (s, 1H), 7.94–7.89 (d, *J* = 9.6 Hz, 1H), 7.63 (t, *J* = 8.2 Hz, 1H), 7.51 (t, *J* = 8.2 Hz, 1H), 6.46 (d, *J* = 9.6 Hz, 1H), 3.47 (q, *J* = 6.3 Hz, 2H), 2.81 (m, 4H), 2.67 (m, 4H), 2.61 (t, *J* = 6.3 Hz, 2H), 2.54 (s, 3H), 1.97 (p, *J* = 6.3 Hz, 2H). ^13^C NMR (400 MHz, CDCl_3_) δ 179.5, 155.6, 153.6, 150.3, 136.5, 133.6, 130.0, 129.5, 129.2, 127.2, 127.1, 126.0, 125.0, 120.5, 114.0, 104.2, 103.1, 55.3, 54.1, 51.5, 44.7, 41.4, 25.5. ( +) ESI QqToF (m/z): Calcd. for C_25_H_27_N_4_O_4_ + : [M + H]^+^ 447.2027, found 447.2024.

##### Data for 4-nitro-1-((3-(pyrrolidin-1-yl)propyl)amino)-12*H*-benzo[*b*]xanthen-12-one (**43**)

Yield: 79%; M.p. 175–176 °C (EtOAc–*n*-Hexane); ^1^H NMR (600 MHz, CDCl_3_) δ 10.73 (t, D_2_O exchang., *J* = 5.3 Hz, 1H), 8.77 (s, 1H), 8.32 (d, *J* = 9.6 Hz, 1H), 8.02 (d, *J* = 8.3 Hz, 1H), 7.99 (s, 1H), 7.93 (d, *J* = 8.4 Hz, 1H), 7.63 (t, *J* = 8.2 Hz, 1H), 7.52 (t, *J* = 8.2 Hz, 1H), 6.50 (d, *J* = 9.6 Hz, 1H), 3.53 (q, *J* = 6.9, 2H), 2.80 (t, *J* = 6.9 Hz, 2H), 2.76 (brs, 4H), 2.10 (p, *J* = 7.0 Hz, 2H), 1.91 (brs, 4H). ^13^C NMR (600 MHz, CDCl_3_) δ 179.8, 155.8, 153.7, 150.5, 136.7, 133.8, 130.2, 129.6, 129.3, 127.4, 127.3, 126.0, 125.2, 120.8, 114.1, 104.4, 103.3, 54.0, 53.4, 41.3, 27.4, 23.5. ( +) ESI QqToF (m/z): Calcd. for C_24_H_24_N_3_O_4_^+^: [M + H]^+^ 418.1761, found 418.1764.

##### Data for 1-((2-morpholinoethyl)amino)-4-nitro-12*H*-benzo[*b*]xanthen-12-one (**44**)

Yield: 82%; M.p. > 220 °C (dec) (EtOAc—*n*-Hexane); ^1^H NMR (400 MHz, CDCl_3_) δ 10.87 (s, D_2_O exchang., 1H), 8.84 (s, 1H), 8.35 (d, *J* = 9.6 Hz, 1H), 8.06 (d, *J* = 8.4 Hz, 1H), 8.02 (s, 1H), 7.94 (d, *J* = 8.4 Hz, 1H), 7.68–7.61 (m, 1H), 7.58–7.48 (m, 1H), 6.45 (d, *J* = 9.6 Hz, 1H), 3.84 (m, 4H), 3.49 (brs, 2H), 2.80 (brs, 2H), 2.61 (m, 4H). ^13^C NMR (400 MHz, CDCl_3_) δ 179.8, 155.7, 154.0, 150.7, 136.8, 134.0, 130.4, 129.8, 129.4, 127.7, 127.5, 126.2, 125.5, 121.1, 114.3, 104.8, 103.6, 67.2, 56.4, 53.6, 40.2. ( +) ESI QqToF (m/z): Calcd. for C_23_H_22_N_3_O_5_^+^: [M + H]^+^ 420.1554, found 420.1560.

##### Data for 1-((2-(4-methylpiperazin-1-yl)ethyl)amino)-4-nitro-12*H*-benzo[*b*]xanthen-12-one (**45**)

Yield: 68%; M.p. > 220 °C (dec) (EtOAc–*n*-Hexane); ^1^H NMR (600 MHz, CDCl_3_) δ 10.83 (t, D_2_O exchang., *J* = 4.8 Hz, 1H), 8.81 (s, 1H), 8.34 (d, *J* = 9.5 Hz, 1H), 8.05 (d, *J* = 8.3 Hz, 1H), 8.01 (s, 1H), 7.94 (d, *J* = 8.4 Hz, 1H), 7.64 (t, *J* = 8.2 Hz, 1H), 7.53 (t, *J* = 8.2 Hz, 1H), 6.43 (d, *J* = 9.6 Hz, 1H), 3.48 (q, *J* = 5.8 Hz, 2H), 3.02–2.74 (m, 10H), 2.57 (s, 3H). ^13^C NMR (400 MHz, CDCl_3_) δ 180.0, 155.9, 154.2, 151.0, 137.1, 134.2, 130.6, 130.0, 129.7, 127.8, 127.8, 126.5, 125.8, 121.3, 114.5, 105.1, 103.8, 55.7, 54.9, 51.6, 45.3, 40.6. ( +) ESI QqToF (m/z): Calcd. for C_24_H_25_N_4_O_4_^+^: [M + H]^+^ 433.1870, found 433.1870.

### Microorganisms strains and growth conditions

The following reference and clinical fungal strains were used: *C. albicans* ATCC 10231, *C. glabrata* ATCC 90030, *C. krusei* ATCC 6258, *C. parapsilosis* ATCC 22019, *S. cerevisiae* ATCC 9763, *C. albicans* B3, *C. albicans* B4, *C. albicans* Gu4, *C. albicans* Gu5, *C. albicans* F2, *C. albicans* F5^27,28^. Fungal strains used in this investigation were routinely grown over 18 h at 30 °C in YPG liquid medium (1% yeast extract, 1% peptone, 2% glucose) in a shaking incubator. For growth on solid media, 1.5% agar was added to the YPG medium.

### Antifungal activity assays

Antifungal in vitro activity was determined by the modified M27-A3 specified by the CLSI^29^ by minimal inhibitory concentrations (MICs) determination as described previously^14^. The MIC was defined as the lowest drug concentration at which at least a 90% decrease in turbidity, in comparison to the drug-free control, was observed. Antifungal activity was determined in RPMI-1640 medium buffered to pH 7.0. The final concentration of the compound solvent (DMSO) did not exceed 2.5% volume of final suspension in each well and did not influence the growth of microorganism.

Minimum fungicidal concentrations (MFCs) were determined as described previously^13^ by spot assay. The MFC was determined as the lowest concentration of the test compound in which no recovery of microorganisms was observed.

### Yeast topoisomerase II relaxation assay and inhibition

The inhibition of yeast topoisomerase II was analysed according to the relaxation assay kit from Inspiralis (Inspiralis Limited, Norwich, UK) and as described previously^14^. The relaxation inhibition effectivity (IC_50_) of the analyzed compounds was determined by densitometry quantification of the transition from supercoiled to relaxed forms and was expressed in relation to the control. The gels were photographed with Gel Doc XR + Gel Documentation System (Bio-Rad: Hercules, CA, 647 USA) and image processing was performed by GIMP 2.10.18.

### Antiproliferative activity determination

The human embryonic kidney cell line (HEK-293) and human liver cancer cell line (HEPG2) were purchased from ATCC (Manassas, Virginia, USA). HEK-293 was cultured in Dulbecco’s Modified Eagle Medium and HEPG2 in Minimum Essential Medium Eagle. For each cell line, the culture medium was supplemented with 10% fetal bovine serum, 2 mM l-glutamine, and antibiotics: penicillin 62.6 μg mL^−1^ and streptomycin 40 μg mL^−1^. The cells were cultured at the humidified atmosphere of 5% CO_2_/95% air and routinely tested for *Mycoplasma* contamination. Antiproliferative activity of the compounds was determined as previously described by MTT method^14^. Percentage of cell viability was calculated, and EC_50_ values were determined using GraphPad Prism^®^ software (version 8.3.1).

## Conclusions

28 xanthone and benzoxanthone analogues were synthesized and evaluated for their antifungal activity. Benzoxanthones proved to be the best antifungal agents with positive mycostatic selectivity index values in relation to HEK293 and HEPG2 cell lines. The fused benzene ring is thus crucial for their activity. The evaluation of biological properties suggests that the mode of action of the most effective derivatives is fungicidal. The demonstrated killing activity is associated with a higher probability of early therapeutic success and a decreased probability of resistance development. Moreover, the clinical strains of *C. albicans* resistant to fluconazole, due to the FLU-induced overexpression of Cdr1p/Cdr2p drug efflux pumps as well as Mdr1p membrane transport protein of the major facilitator superfamily (MFS), remained sensitive to our novel compounds. This finding suggests that the most active against clinical resistant strains compounds **9**, **42** and **44** are not good substrates for ABC as well as MFS efflux pumps.

Since xanthone derivatives are human topoisomerase inhibitors, the antifungal activity of analyzed compounds may be related to their inhibitory effect on the fungal equivalent of that enzyme. Our results indicate a strong relationship between the antifungal activity and the inhibitory effectiveness against yeast topoisomerase II.

Summing up, we were able to show a proof of concept that xanthone modification may result in the discovery of a new group of selective antifungal drugs affecting fungal topoisomerase II. Moreover, our results indicate the possibility of using those derivatives against resistant fungal cells. Further validation of xanthones applicability as antifungals by designing, synthesizing and evaluating the activity of new inhibitors is highly valuable. Future investigations will be focused on improving selectivity.

## Supplementary Information

Supplementary Figure S1.

## Data Availability

All data generated or analysed during this study are included in this published article.
