# Supplementary material for: Xanthone synthetic derivatives with high anticandidal activity and positive mycostatic selectivity index values
Source: Sci Rep. 2023 Jul 23;13:11893. doi: 10.1038/s41598-023-38963-4 (PMC10363532; doi:10.1038/s41598-023-38963-4)
Supplement: Supplementary file 1 — Supplementary Figure S1. [file 41598_2023_38963_MOESM1_ESM.pdf]

Xanthone synthetic derivatives with high anticandidal activity and positive mycostatic selectivity index values

Kamila Rząd[1], Rachel Ioannidi[2], Panagiotis Marakos[2], Nicole Pouli[2], Mateusz Olszewski[1], Ioannis K. Kostakis[2], Iwona Gabriel[1]*

[1]Department of Pharmaceutical Technology and Biochemistry, Faculty of Chemistry and BioTechMed Center, Gdańsk University of Technology, 11/12 Narutowicza Str. , 80-233 Gdańsk, Poland

[2]Division of Pharmaceutical Chemistry, Department of Pharmacy, School of Health Sciences, National and Kapodistrian University of Athens, Panepistimiopolis, 15771 Zografou. Greece.

* Corresponding author. Tel: +48 583486078; Fax: +48 583471144; E-mail: iwona.gabriel@pg.edu.pl

Supplementary Figure S1. Full length gels for Figure 4

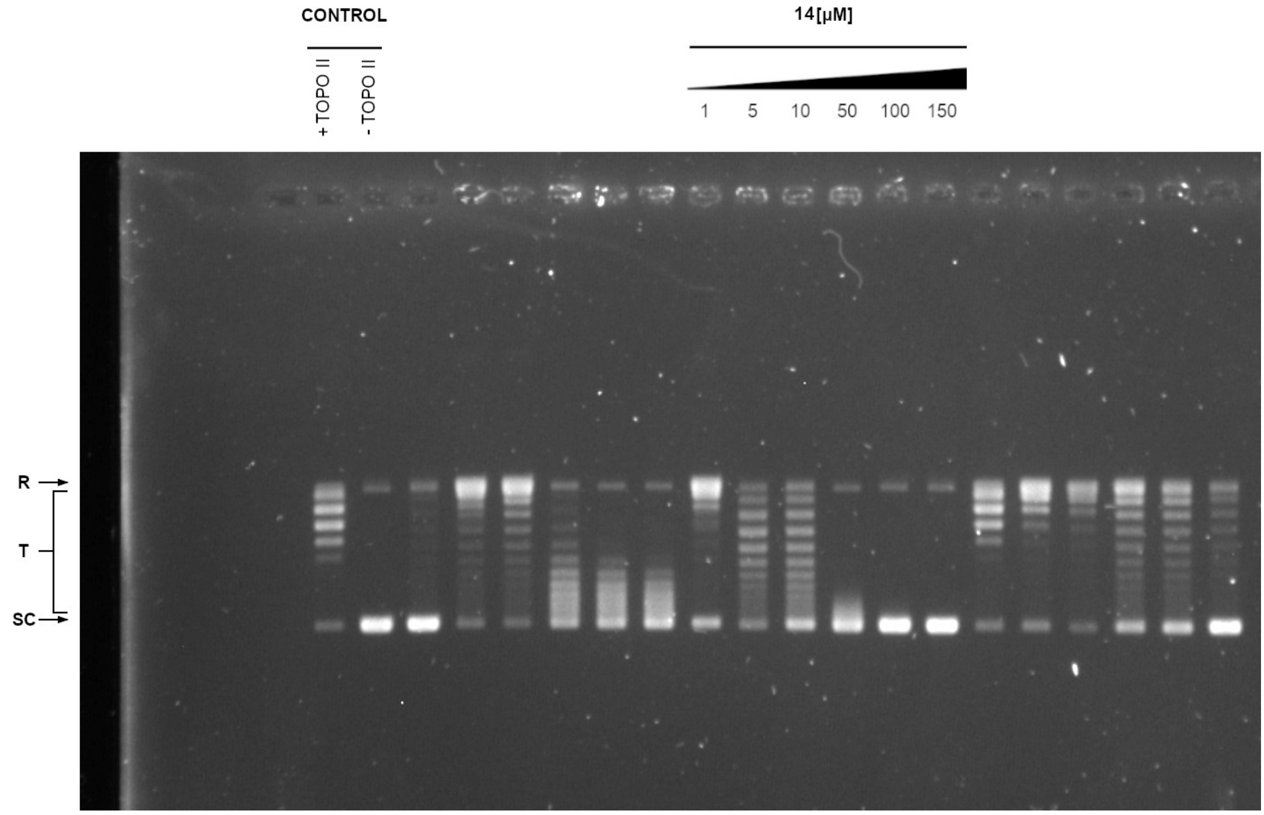

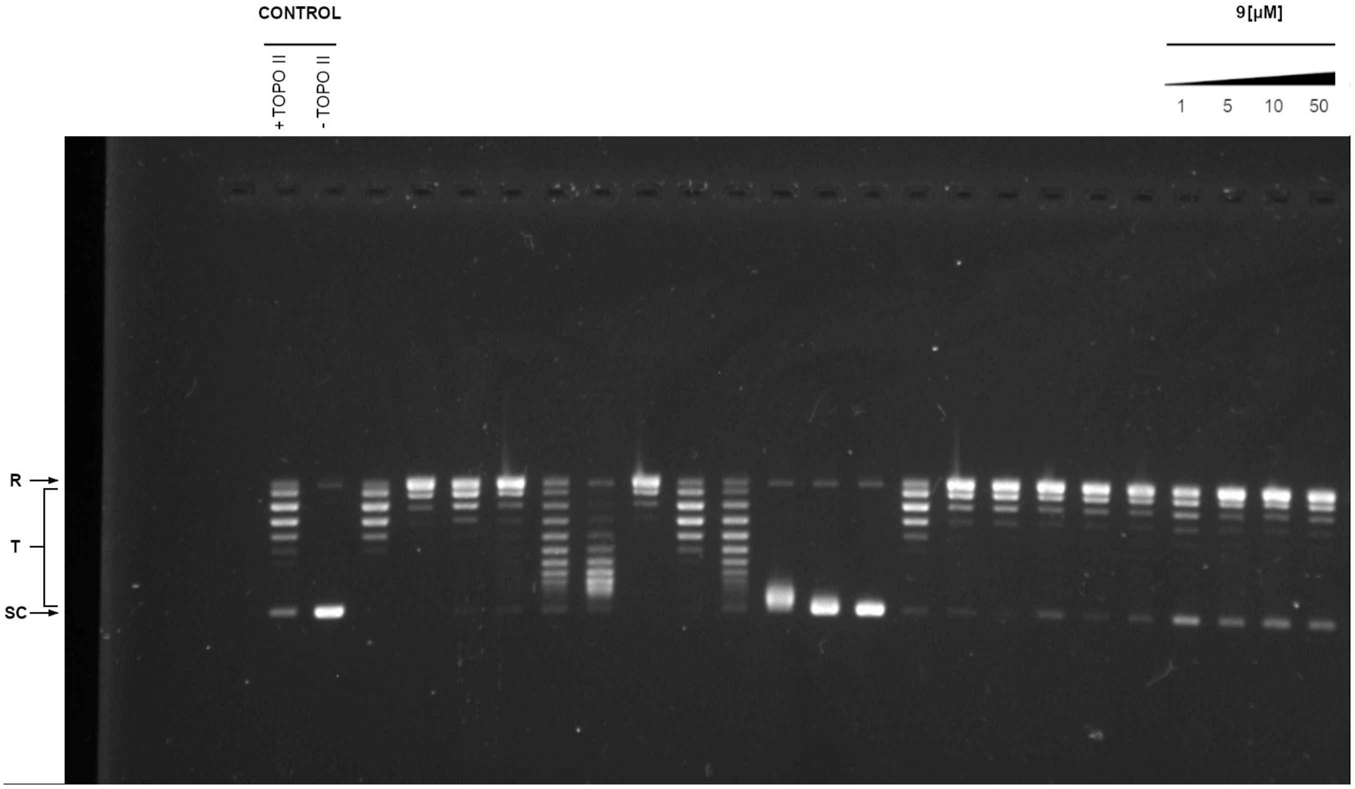
CONTROL
+ TOPO II
- TOPO II
9[µM]
1
5
10
50
R
T
SC

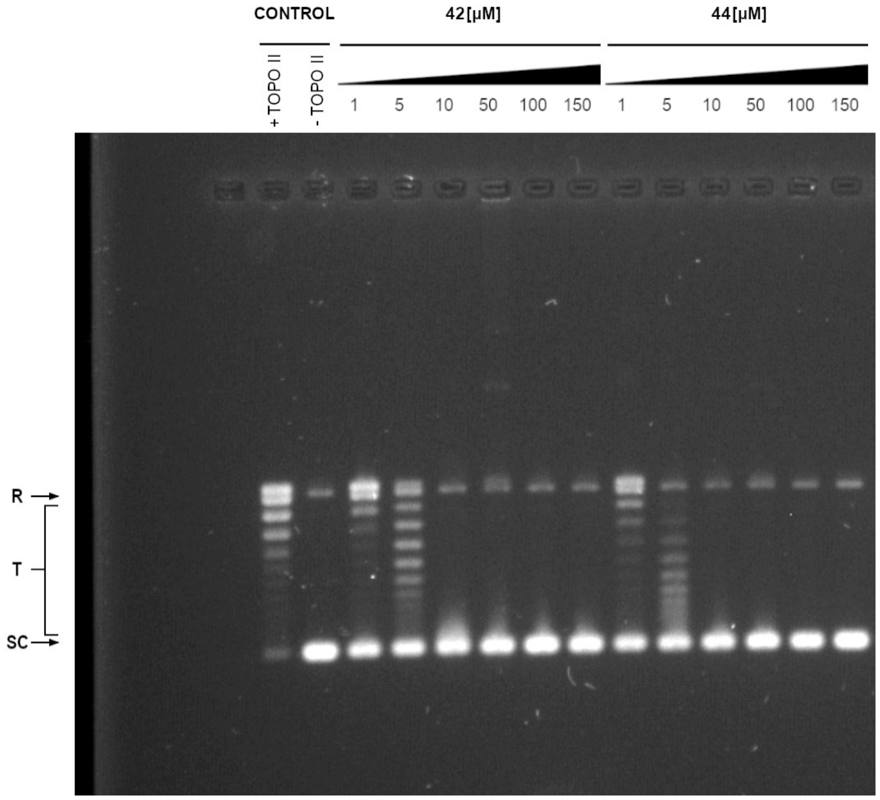
CONTROL
+ TOPO II
- TOPO II
42[µM]
1
5
10
50
100
150
44[µM]
1
5
10
50
100
150
R
T
SC
